# Enhancing Response Surface Methodology for Bioprocesses Development Using Interpretable Machine Learning

**DOI:** 10.1021/acsomega.6c09217

**Published:** 2026-10-29

**Authors:** Juan Federico Herrera-Ruiz, Juan Camilo Castaño-Sánchez, Oscar Andrés Prado-Rubio

**Affiliations:** † Grupo de Investigación en Aplicación de Nuevas Tecnologías (GIANT) Departamento de Ingeniería Química, Universidad Nacional de Colombia sede Manizales, Campus La Nubia, Manizales 170003, Colombia; ‡ TBI, Université de Toulouse, CNRS, INRAE, INSA, 135 Avenue de Rangueil, Toulouse (CEDEX 04) 31077, France; § Grupo de Investigación en Aplicación de NuevasTecnologías (GIANT) Departamento de Ingeniería Química, Universidad Nacional de Colombia sede Manizales, Campus La Nubia, Manizales 170003, Colombia

## Abstract

Response surface methodology (RSM) has been a cornerstone for experimental bioprocess optimization, yet its reliance on polynomial equations often fails to capture complex nonlinearities and requires extensive experimental data to attempt capturing them. This study evaluates the enhancement of RSM through machine learning (ML) techniques, specifically Gaussian Processes and Support Vector Machines, across three biological systems: succinic acid fermentation, biobutanol production, and continuous ethanol cultivation. To mitigate the “black-box” nature of ML, explainability measures such as Mutual Information (MI) and Local Interpretable Model-agnostic Explanations (LIME) were integrated to assess feature importance and ensure biological relevance. Results demonstrate that ML models consistently outperform traditional polynomials in predictive accuracy, even when having access to less experimental data for calibration and a reduced space of features, particularly on validation data sets where errors were significantly reduced (e.g., reaching MAPE lower than 1% in simulated experimental scenarios). Furthermore, the application of Model-Based Design of Experiments using Latin Hypercube Designs achieved optimal conditions with up to 50% fewer experimental runs compared to traditional factorial approaches. These findings suggest that coupling ML with explainability tools improves the reliability of finding optimal conditions for bioprocess development while also significantly enhancing operational efficiency by reducing experimental runs, time, and costs.

## Graphical abstract

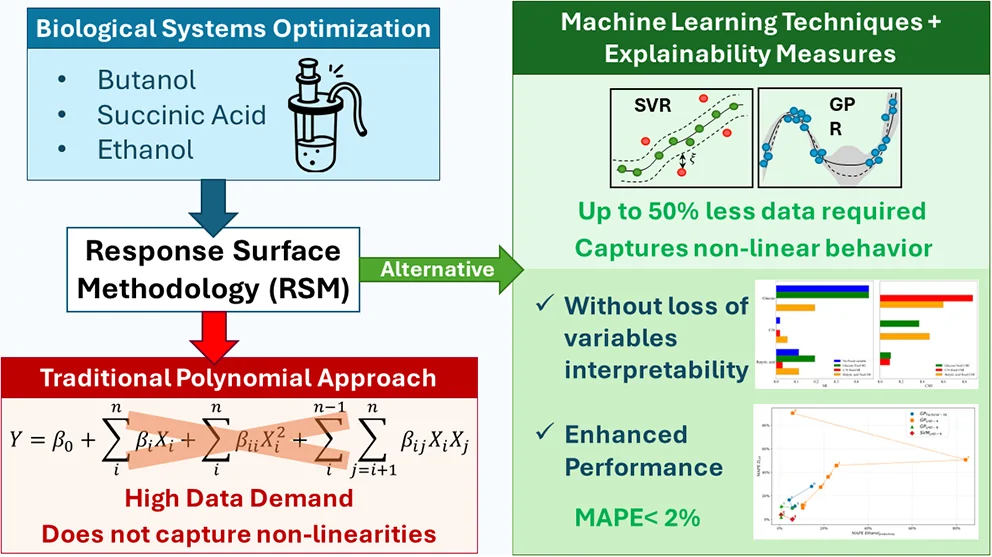

## 1 Introduction

Response surface methodology (RSM) is a set of statistical and mathematical techniques commonly used to model, analyze, and optimize a process or phenomenon of interest in an early stage of development.^1^ It aims to describe one or several responses influenced by multiple independent variables. Due to the complexity of modeling certain phenomena or processes, RSM has been applied in different fields, including food industry processes,^2^ bioenergy,^3^ biotechnology,^4^ bioprocesses^5–8^ water treatment,^9–11^ combustion engineering,^12^ analytical chemistry,^13^ among many others.

In process engineering and bioprocesses, RSM has been widely used to describe and optimize, in an early stage, the bioreactor’s behavior.^6,7,14,15^ This methodology is particularly useful because it allows the identification of the correlation between independent variables (e.g., temperature, pH, substrate concentration, aeration rate, agitation, among others) and response variables, which many phenomena-based models cannot incorporate into their equations when the kinetics and transport phenomena have not been investigated in detail.^16^ Moreover, the development of phenomenological or kinetic models in the preliminary phases of process development can often be tedious or impractical due to the limitations in measuring specific intermediate compounds within complex metabolic pathways, as well as the lack of knowledge of the intrinsic behavior of the system.^17^

The implementation of RSM involves five main steps:^3,4,12^

i. Identification of independent input factors that affect the response variables.
ii. Selection and application of an appropriate Design of Experiments (DoE) to efficiently collect data through experimental runs.
iii. Mathematical fitting and evaluation of a model obtained from experimental data.
iv. Interpretation of the response model and/or optimization of the response variable(s) based on the fitted models.
v. Validation of the predicted response variable(s) by performing verification experiments.

Despite this systematic approach, several limitations arise in practice. For instance, increasing model robustness (by considering more factors or levels) leads to an exponential growth of the number of experiments required for factor identification and DoE implementation, thereby reducing its efficiency.^13,18^ This, in turn, raises the costs associated with reagents, equipment, energy, and personnel availability. Furthermore, nonlinearity of some response variables, such as microbial growth, substrate uptake, or metabolite production over time, also limits the RSM models’ capabilities. These response variables often show asymptotic sigmoidal behavior, which is difficult for polynomial model fitting, leading to poor prediction of optimal conditions.^16,19^

To address these limitations, recent studies have compared and incorporated machine learning (ML) techniques with DoE,^7,8,14,17,20,21^ being the most common used techniques Artificial Neural Networks (ANN) coupled with Genetic Algorithms (ANN-GA) for optimization. These approaches capture nonlinearities more effectively than the polynomial models used in RSM.^14,16,21^ Although ANN-based models have been widely adopted over the last 2 decades,^14^ its *ad-hoc* selection is not sufficiently justified and alternative ML techniques such as Gaussian Processes (GPs) and Support Vector Machines (SVMs) remain less explored in bioprocess modeling These techniques often require less experimental data, reduce computational effort, and are less prone to overfitting when applied to small data sets, as seen in hydrology applications,^22^ medical screening,^23^ hybrid modeling of succinic acid fermentation,^24^ and material sciences, nuclear physics and cosmology.^25^

Despite the ML potential, their applicability is hindered by model interpretability. Polynomials have the advantage of being explainable by design, that is, researchers can understand how and why the features affect the outcomes,^26,27^ perceptible directly from the coefficients of the polynomials. Traditionally, ML models are deemed not interpretable; however, several explainability techniques have been developed to strengthen ML models’ interpretability, most notably *post*-*hoc* interpretability such as SHAP values,^28^ ceteris paribus^29^ or LIME.^30^ These techniques have been applied to fields such as chemistry,^31^ materials science,^32^ metallurgic industry,^33^ hybrid modeling of butanol and therapeutic protein production,^34,35^ and plant-wide chemical processes.^36^ To the best of our knowledge, these techniques have not been applied to pure ML models describing biological systems. In this work, we aim to show how ML models such as GPs and SVMs, coupled with feature selection techniques and explainability measures, can enhance RSM applied in different contexts for early-stage bioprocess development.

For this purpose, we propose a computational proof of concept in which three different scenarios are used to assess how the combination of these techniques can enhance RSM performance by improving the prediction of optimal operating conditions by (i) using a reduced set of features (factors) while retaining the same experimental data; (ii) employing a reduced set of experimental data; and (iii) using fewer experimental runs. Therefore, herein it is illustrated how the combination of techniques could, in principle, strengthen the models’ descriptive and predictive capabilities while potentially reducing experimental runs, thus reducing time and related costs.

Figure 1 presents the highlights of the investigated case studies, depicting the experimental design status, how the approach was used for different purposes, and the obtained outcome.

**Figure 1:**
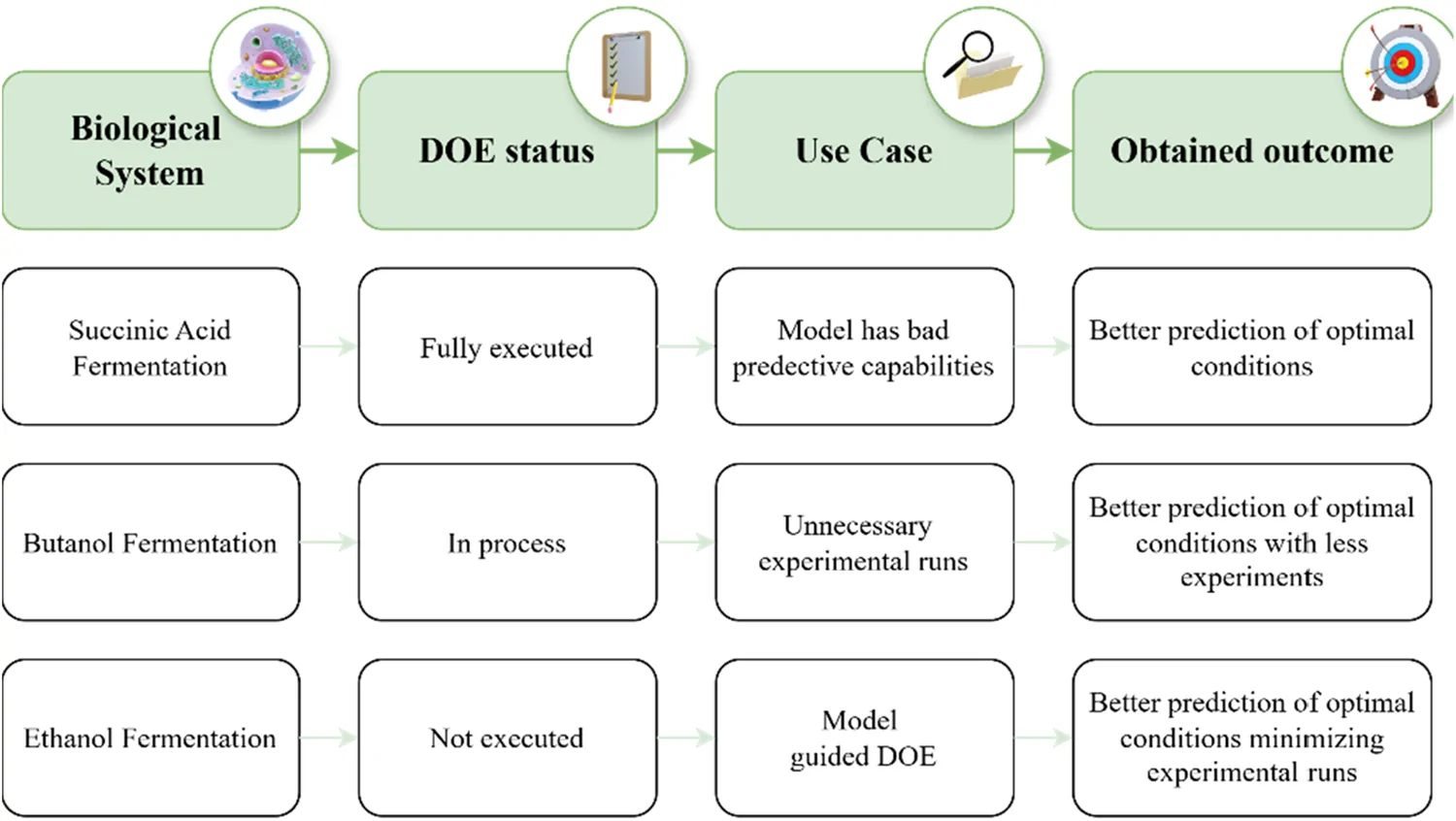
Case study highlights.

The first case study focused on succinic acid fermentation. In this case, data was taken from the literature for *Actinobacillus succinogenes*.^37^ Authors studied the effect of initial glucose concentration, yeast extract, and inorganic carbon concentration on final succinic acid production. Fermentations were carried out for 12 h. Despite good performance indexes on calibration experiments (*r* ^2^ > 0.9), prediction on validation conditions remains poor, with errors higher than 30%. As such, we investigated the potential use of SVMs and GPs to enhance prediction on validation experiments once the full DoE has been executed.

Butanol production using *Clostridium acetobutylicum* was selected as the second case study. In this research, the authors investigated the effect of glucose concentration, carbon/nitrogen ratio, and initial butyric acid concentration, with experimental runs taking more than 140 h.^38^ We aimed to assess whether it was necessary to fully perform the DoE, or if a reduced set of experiments sufficed to maintain prediction quality. As such, we systematically removed experiments from the calibration of ML models and evaluated their performance on validation experiments and tested the prediction quality on the optimized conditions. This was done to evaluate ML models’ capabilities of maintaining prediction performance when using less available data than the full polynomial models.

Continuous ethanol production by *Saccharomyces cerevisiae* was investigated to find the multiplicity of steady-state conditions and critical dilution rate.^39^ Experimental runs to achieve steady-state operations took up to 90 h. This final case study uses the proposed model to generate synthetic data and test ML models’ predictive capabilities in a real setting. ML models were trained on preliminary data and used to assess whether a model-based DoE approach using ML models could reduce experimental runs to find optimal conditions when compared with RSM polynomial models.

This paper is structured as follows: Section 2 introduces the general methodology, detailing the feature selection and interpretability techniques. Section 3 presents the main results of applying ML models to bioprocesses. Finally, Section 4 contains conclusions and future perspectives.

The main contributions of this study are that ML models can have equal or better performance than traditional polynomial models in RSM. Moreover, ML models had superior performance in calibration (training and testing) and validation sets while using less information than the polynomial models calibrated. Finally, interpretability techniques allowed us to draw insights from the ML models’ outputs, helping us understand how ML models make predictions.

## 2 Methodology

### 2.1 General Methods

Figure 2 presents the general methodology followed for each case.

**Figure 2:**
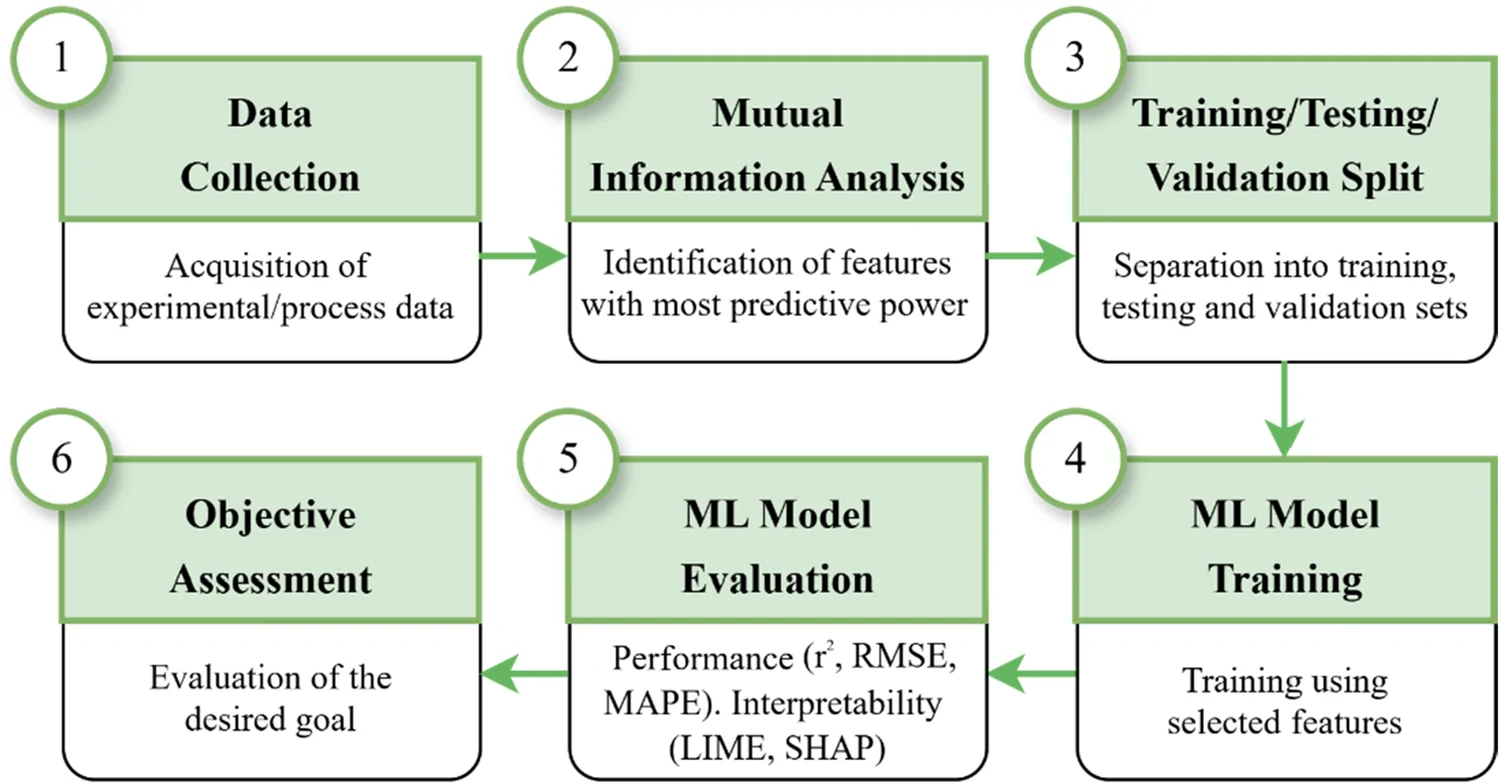
Proposed framework for the case studies.

First, data was collected from different sources,^37,38^ whereas kinetic parameters were taken for the ethanol production case.^39^ After, Mutual Information analysis^40^ was used for feature engineering, aiming to assess which experimental features contain predictive power before training ML models. The data points were then split into calibration (training and testing) and validation sets, ensuring that every level of the experimental factors was present in the validation set. Inside the calibration data set for Cases 1 and 3, an 80/20 split was used for training and testing, respectively, to allow for hyperparameter optimization. Subsequently, ML models were trained, and their performance was evaluated using error metrics (RMSE, MAPE, or *r* ^2^) with the complete feature set and then a subset of features, in order to assess the effect of feature selection and reduced input information. The results were further complemented by interpretability techniques to improve ML model understanding. Finally, an evaluation regarding the goal of each case was performed.

### 2.1.1 Mutual Information Analysis

Mutual Information (MI) quantifies the relationship between possible features and the desired outputs, enabling investigation of nonlinear relationships and assessment of how uncertainty of possible inputs relates to the desired outputs’ uncertainty.^40^ MI enables assessment of nonlinear relationships and multiple inputs for a desired target, unlike Pearson’s correlation.^41,42^ MI is defined as eq ([Disp-formula eq1]).

1 I(X;Y)=H(X)+H(Y)−H(X,Y)

where *X* and *Y* denote continuous input and output variables, respectively, *H­(X)* represents the entropy of variable *X*, defined in eq ([Disp-formula eq2]), *H­(X,Y)* represents the joint entropy for variables *X* and *Y*, defined in eq ([Disp-formula eq3]). *I­(X;Y)* measures how much knowing *X* reduces uncertainty about *Y*. As such, if *I­(X;Y)=*0 both variables are independent. On the other hand, larger values of *I­(X;Y)* show its dependency.^42^

2 H(X)=−∫p(x)·log⁡p(x)dx

3 H(X,Y)=−∬p(x,y)·log⁡p(x,y)dxdy

where *x* is a specific value of *X* and *p­(x)* is a probability density function of *X*.

Finally, Conditional Mutual Information (CMI) allowed evaluation of extra information gained when adding specific variables as inputs to the ML models. As such, CMI is calculated as seen in eq ([Disp-formula eq4]):

4 I(X;Y|Z)=H(X,Z)+H(Y,Z)−H(Z)−H(X,Y,Z)

where *H­(X,Y,Z)* is defined as seen in eq ([Disp-formula eq5]):

5 H(X,Y,Z)=−∭p(x,y,z)·log⁡p(x,y,z)dxdydz

The higher the CMI values, the more extra information is provided by *X* about *Y*, considering that input *Z* is already known.^41^

MI and CMI were estimated using the NPEET library employing continuous distributions and a 3-nearest neighbor estimator.^43^ To minimize variability of the practical MI and CMI calculation, values reported are the average of 100 runs using the full experimental data set and using data shuffling.

### 2.2 Training Machine Learning Models and Performance Assessment

For this study, SVMs and GPs were used as ML Models. Due to the stochastic nature of their calibration, 1000 ML models were calibrated for each case, and performance was assessed on the validation set. As such, the best model for each technique was used for comparison against the conventional polynomial models. Performance was ranked using eq ([Disp-formula eq6]):

6 $$\begin{array}{l} \min\left({p\mathrm{rediction}}_{quality}+o\mathrm{verfitting}\right)\\ {p\mathrm{rediction}}_{quality}=\left({NRMSE}_{\mathrm{train}}+{NRMSE}_{\mathrm{valid}}\right)\\ o\mathrm{verfitting}=\left|{NRMSE}_{\mathrm{train}}-{NRMSE}_{\mathrm{valid}}\right| \end{array}$$

with NRMSE being RMSE normalized by the range of experimental data.

### 2.2.1 Explainability of Machine Learning Models

One of the main advantages of polynomial models is that each coefficient can be explained and assigned physical meaning toward the prediction, whereas machine learning models usually are “black-boxes” regarding the relationships between inputs and outputs.^44^

Logical Interpretable Model-agnostic Explanations (LIME) was chosen to explain the ML outputs. LIME takes a specific ML model and generates synthetic data around a specific point in space by making perturbations to the input features.^30^ Then, a sparse linear model is trained to reproduce ML models’ predictions around the specified point, but not necessarily globally.^30^ As such, after choosing the best ML model for each case, LIME tests were performed to interpret how ML models used its input features.

### 2.3 Case 1: Enhancing Prediction on Unseen Data

The authors performed a three-factor, five-level Central Composite Design of experiments (CCD), comprising 20 experimental points.^37^ Initial glucose concentration, yeast extract, and MgCO_3_ concentrations were used as factors for succinic acid production by *A. succinogenes*. After the CCD was performed, 4 additional experimental runs were used as confirmation for model validity, with each fermentation taking around 17 h. The authors did not report optimal conditions.

As a test, the proposed ML models were trained on the initial CCD points, and the 4 validation experiments were used as a validation set for assessing performance.

### 2.4 Case 2: Reducing Experimental Runs for Bioprocess Optimization

Butanol production by *C. acetobutylicum* was analyzed using a CCD with 20 experimental points. Each experimental run took up to 160 h.^38^ The polynomial model approach was used for finding optimal conditions.

For the case study evaluation, 4 experiments comprising all the different levels for the factors were selected for validation, and ML models were trained on the remaining data. Once the best ML model was found, performance on the predicted optimal conditions was assessed (testing).

Subsequently, one experiment from the calibration data set was removed and added to the validation set, repeating training, validation, and testing with the predicted optimal conditions. This was done for all the experiments in the calibration data set, and the experiment that yielded the best performance was permanently added to the validation data set. This procedure was repeated up to five times. This reduced the calibration data sets from 16 to 11 experiments. This allowed the evaluation of ML models’ capabilities for prediction when using less available data than the polynomial approach using the full data set.

### 2.5 Case 3: Model-Based DoE

In this case, a structured kinetic model for the aerobic ethanol cultivation using *S. cerevisiae* was used to generate synthetic data.^39^ The authors investigated the Crabtree effect for *S. cerevisiae* in continuous operation (*overflow metabolism*). Ethanol productivity increases, reaching its peak at the critical dilution rate, and further increases in dilution rate result in decreases in ethanol productivity, due to bioreactor washout. This system is interesting since the cultivation can oscillate due to the presence of stable and unstable steady states at intermediate dilution rates, making the system complex, both from experimental and simulation perspectives.

The kinetic model was used to simulate synthetic experimental runs. As such, the model was solved at different feeding substrate concentrations and dilution rates to generate synthetic data on all 8 states, focused on maximizing ethanol productivity. This data was used to fit ML models and perform a sequential model-based DoE (MBDoE). In sequential MBDoE approaches, an initial set of experiments is performed, and the desired output is selected for optimization.^45^ The model is used to find optimal conditions, and the experiment is performed.^46^ This process is repeated until the prediction agrees with the experimental outcome.^47^

As such, for generating the initial data points, two different DoEs were tested: Latin hypercube design (LHD) and factorial designs. Initial data sets consisted of 4, 9, and 16 data points, with feeding substrate concentration between 30 and 60 g/L and Dilution Rate between 0.35 h^–1^ and 0.5 h^–1^. LHD presents advantages over traditional Factorial Designs in terms of flexibility, data density, and noncollapsing and optimizable properties for exploring the feature space.^48^ After the initial synthetic data was generated, an MBDoE was tested using both ML algorithms and the conventional polynomial fit. Optimal conditions were obtained by using Bayesian optimization, as it has shown promising results for bioprocesses when stochastic models are used.^49^ Bayesian optimization utilizes probabilistic black-box models (usually Gaussian Processes) and acquisition functions to search for optimal solutions in the explored region of the design space (usually called exploitation) and in the unexplored regions (usually called exploration).^49,50^

To evaluate models’ performance, it was assessed whether they correctly found the critical dilution rate and the maximal ethanol productivity obtained by the original parametric model at those conditions, which has been validated experimentally.

### 2.6 Computational Details

All computations were performed on a commercial laptop with an Intel-i7–10510U CPU 1.80 GHz, with 4 cores, 8 GB of RAM. Model calibrations and evaluation were performed in MATLAB 2024a, and Python 3.11.1 was used for Feature Engineering analysis.

## 3 Results and Discussion

All computations took around 2 min for Cases 1 and 2, while up to 2 h for Case 3, due to the need to solve the ODE system repeatedly. Additional computational details can be found in Supporting Information.

### 3.1 Case 1: Enhancing Prediction on Unseen Data

To enhance final succinic acid concentration in *A. succinogenes* fermentation, the authors explored glucose, yeast extract, and MgCO_3_ concentrations as input features, with Table S1 containing the experimental data as originally reported.^37^ For the preprocessing stage, Figure 3 presents MI and CMI indexes for the feature selection used for exploring succinic acid production. On the left, MI is presented for both singular features and combinations of features. As seen in the blue bars, Yeast Extract contains more information on the final succinic acid concentration, whereas MgCO_3_ contains the least, with Glucose having 16% more MI than MgCO_3_.

**Figure 3:**
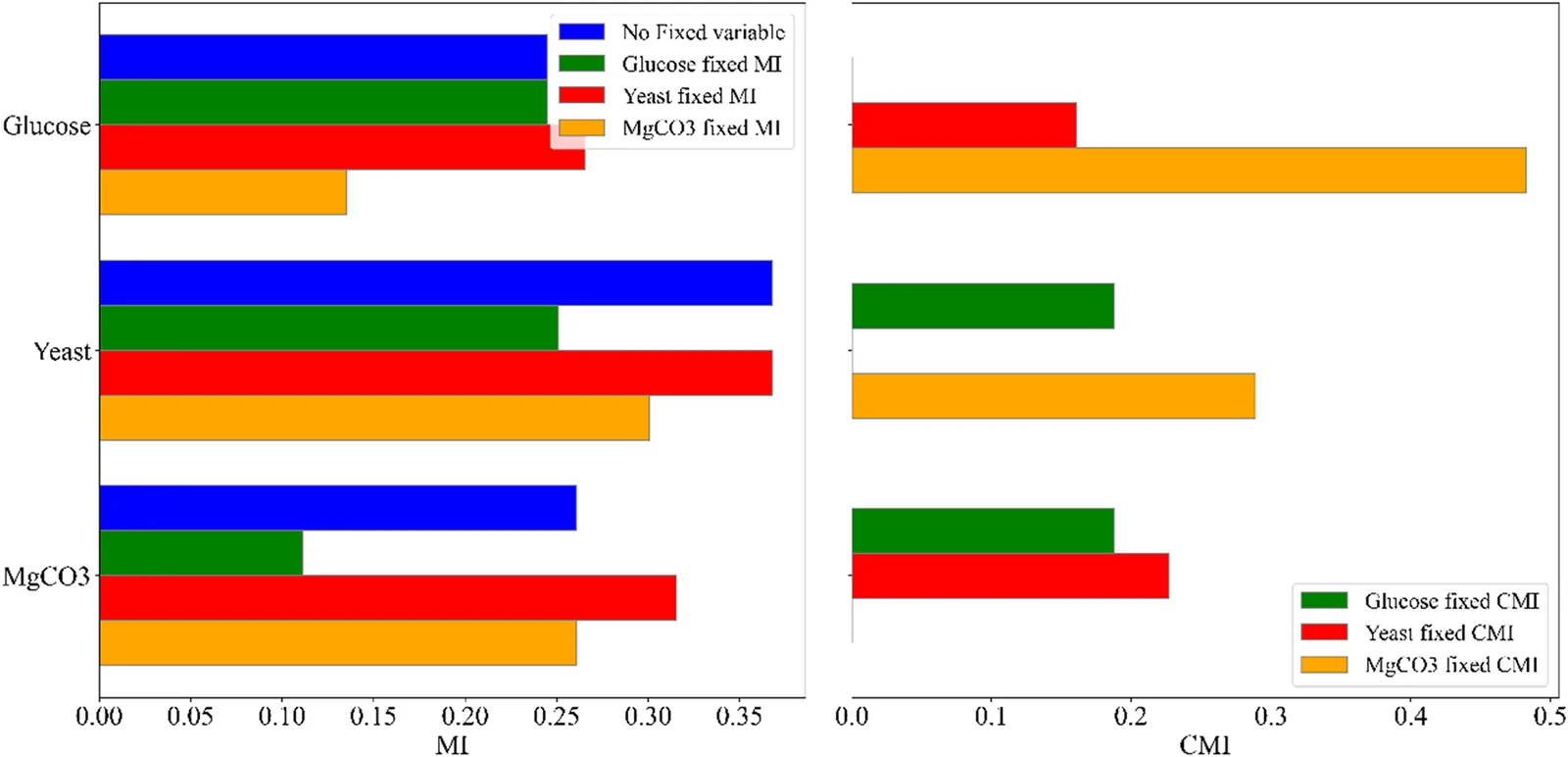
Mutual information (left) and conditional mutual information (right) of the different factors explored in the succinic acid fermentation.

Once yeast extract concentration is fixed as an input, the inclusion of glucose or MgCO_3_ decreases its MI on the target variable; this reduction could be interpreted as the new variables introducing numeric noise to the predictor, which should be interpreted as this variable having no relevant statistical information on the desired target.^41–43^ However, MI’s decrease is slightly less drastic when it is fixed glucose than MgCO_3_. Conversely, fixing yeast extract to glucose or MgCO_3_ concentration increases their MI, suggesting that both variables have better predictive power when coupled with yeast extract. In contrast, MgCO_3_ might introduce additional noise to the predictor when it is coupled with Glucose (and vice versa), as suggested by a decrease in the MI of both features. These results indicate that MgCO_3_ is statistically redundant once glucose and yeast extract are known.

On the right side of Figure 3, CMI is presented. These bars should be interpreted as the additional MI one feature has on the target variable, once the fixed feature is known. These results also indicate that MgCO_3_ contains little unique information on succinic acid’s final concentration, as the other two features have more unique information (length of blue bars) than the one MgCO_3_ adds. Interestingly, glucose concentration contains less unique information than yeast extract (see length of green and red bars on the right), signaling that the reduction of MI seen on the left side could be interpreted as the feature having no relevant information.

After the previous results analysis, it was decided to test ML models using the three input features and solely glucose and yeast extract. Despite yeast extract alone having the most MI, calibrating a model based solely on one feature ignores the mechanistic system knowledge.

Table 1 presents the calibration and validation performance for the different models analyzed. The coefficient of determination is used only as a comparative metric for calibration data sets, as it fails to capture nonlinear behavior, especially in biological sciences.^51^ For the validation subset, RMSE and MAPE were chosen as performance metrics.

**Table 1:** Training and Validation Performance Metrics for the Different Models

		calibration		validation	
model	features	*r* ^2^	RMSE	MAPE	RMSE
Polynomial	Glucose, Yeast Extract, MgCO_3_	0.868	1.372	4.81%	0.636
GP	Glucose, Yeast Extract, MgCO_3_	0.914	0.918	1.25%	0.260
GP	Glucose, Yeast Extract	0.904	1.025	1.36%	0.285
SVM	Glucose, Yeast Extract, MgCO_3_	0.913	1.007	2.19%	0.368
SVM	Glucose, Yeast Extract	0.903	1.164	3.92%	0.494

As seen, ML models present better performance across both calibration and validation data sets. Even when using less features, prediction quality is not compromised, with no significant performance differences using three features or the reduced feature space.

To understand how each feature is used inside the ML models, LIME was used as a benchmark. Figure 4 shows the average LIME feature importance scores along both calibration and validation data sets for the different feature sets when using the best GP model, as the top-performing model. As seen, GPs rely almost exclusively on Glucose and Yeast Extract contributions, with MgCO_3_ adding little weight to the prediction. All features exhibit positive contributions to the model output, indicating that an increase in any feature value led to a corresponding increase in the predicted value. Moreover, results from Figure 4 confirm what the MI index predicted: that yeast extract is the variable with most information on the target variable. On the other hand, these results agree with the traditional polynomial approach, which indicates that MgCO_3_ is not statistically significant for the prediction, as shown in Table S2 in the Supporting Information. Table S2 also contains the estimated value, *F*-value, and *P*-value for the polynomial terms found by the original authors.

**Figure 4:**
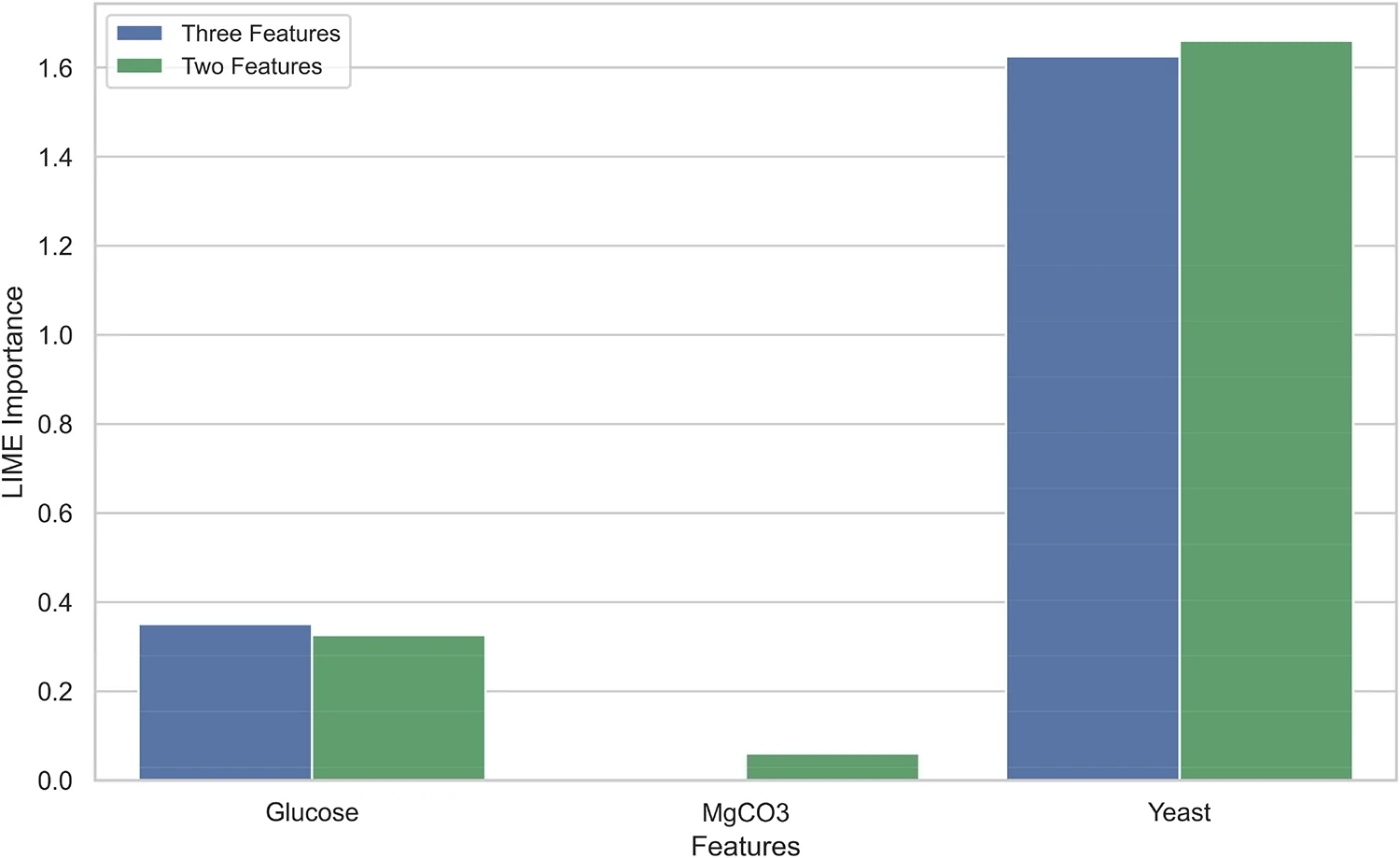
Average LIME importance scores for predicting succinic acid concentrations when using GPs.

The use of the MI + LIME approach presents the advantage of both preserving interpretability of the different features while avoiding statistical artifacts, as seen by the polynomial model assigning significance to the interactions between Yeast Extract and MgCO_3_ (Table S2). In addition, this approach enables the identification of the features that significantly contribute to the prediction, as observed for the MgCO_3_ feature, which also exhibits no statistical significance in the polynomial model.

In this case study, it has been shown how the ML models can achieve higher performance in predicting succinic acid production in both calibration and validation data sets using the same number of experiments while using less information (one less experimental feature).

### 3.2 Case 2: Reducing Experimental Runs for Bioprocess Optimization

For predicting butanol concentration, three input features were investigated.^38^ Figure 5 presents both MI and CMI of the explored variables for predicting butanol. As can be seen in the figure on the left, glucose is the most informative feature for predicting butanol concentration, followed by butyric acid. In contrast, the ratio carbon/nitrogen (C/N) has little information on butanol concentration, and when combined with glucose and butyric acid, it reduces their MI on the desired target, thereby introducing noise to the model prediction. Furthermore, glucose exhibits higher unique information than butyric acid and ratio C/N, as evidenced by CMI values in the figure on the right. Notably, butyric acid exhibits the lowest CMI value, suggesting that the information it provides is redundant and possibly partially captured by the glucose and ratio C/N features.

**Figure 5:**
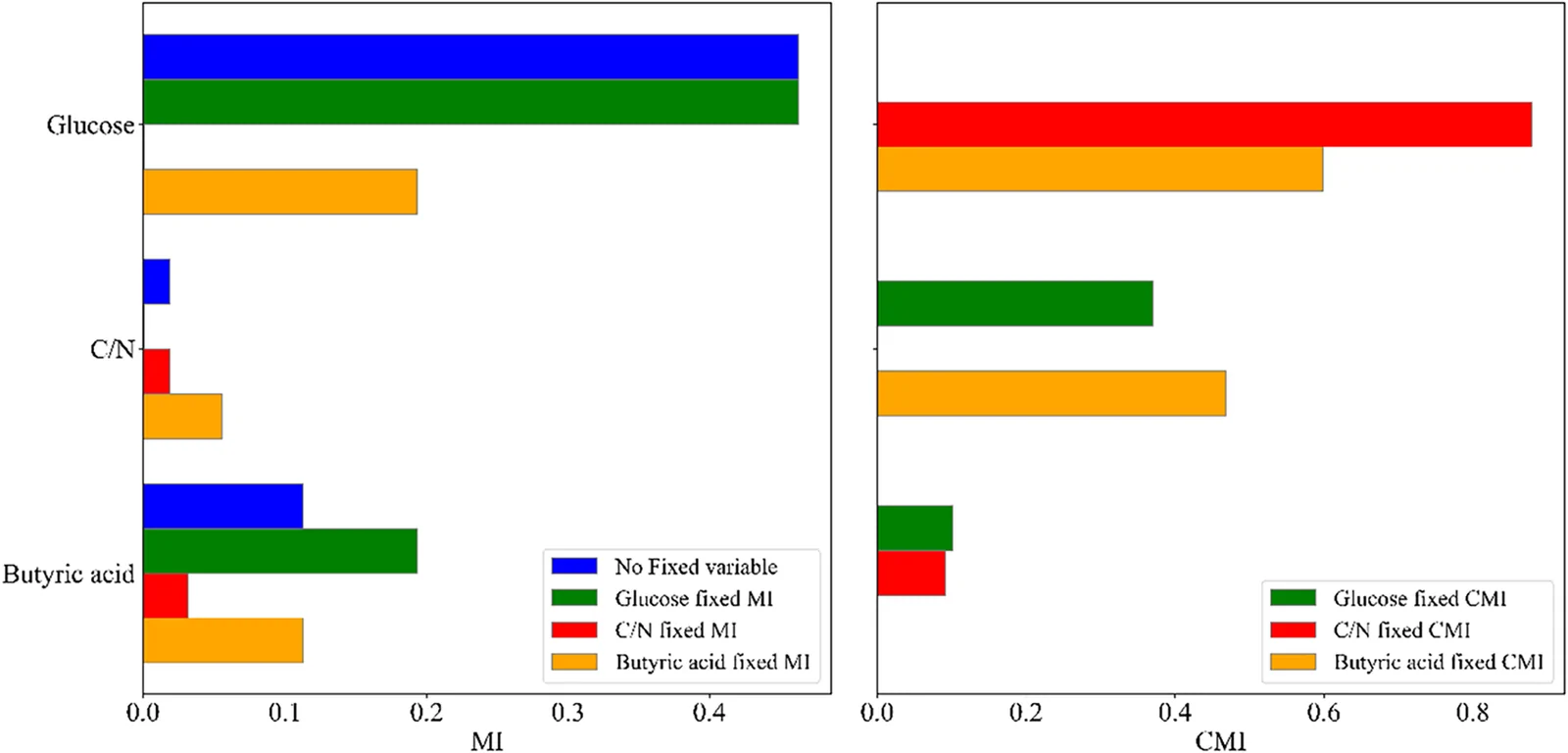
Mutual information (left) and conditional mutual information (right) of the different factors explored in the butanol fermentation.

To assess if every experiment was necessary to maintain model performance, a training/testing split of 16 and 4 experiments was conducted, with the optimal conditions serving as the validation set for the model’s performance. Table S3 presents the experimental conditions as reported by the original authors.^38^ The experiments for testing were chosen to ensure that factors contain all the explored levels in the original CCD design. Table 2 presents the performance of the different models trained on the DoE, reporting the polynomial models’ performance trained on both the full data set and the chosen training experiments. All the ML models performed better across the training/testing and validation, presenting lower RMSE (both training and testing) and MAPE (validation) and higher determination coefficient (training). Interestingly, when removing some experiments from training, the polynomial model presents better performance indexes but suffers from worse generalization capabilities, as seen by higher RMSE in testing and higher MAPE in validation. For fair comparison, the following discussion will use as baseline the Polynomial fitted with the same data as the ML models.

**Table 2:** Training and Testing Performance Metrics for the Different Models Predicting Butanol Performance

		training		testing	
model	features	*r* ^2^	RMSE	MAPE	RMSE
Polynomial^a^	Glucose, C/N, Butyric Acid	0.900	0.626	20.12%	0.972
Polynomial	Glucose, C/N, Butyric Acid	0.924	0.430	32.88%	1.864
GP	Glucose, C/N, Butyric Acid	0.992	0.223	12.01%	0.241
GP	Glucose, Butyric Acid	0.940	0.327	12.08%	0.439
SVM	Glucose, C/N, Butyric Acid	0.965	0.492	12.58%	0.364
SVM	Glucose, Butyric Acid	0.944	0.580	10.18%	0.590

a Performance indexes are calculated using the full data set. The original model does not contain a validation subset, but performance was added to the table for comparison.

Figure 6 presents the average LIME importance for the three different features investigated for butanol production.

**Figure 6:**
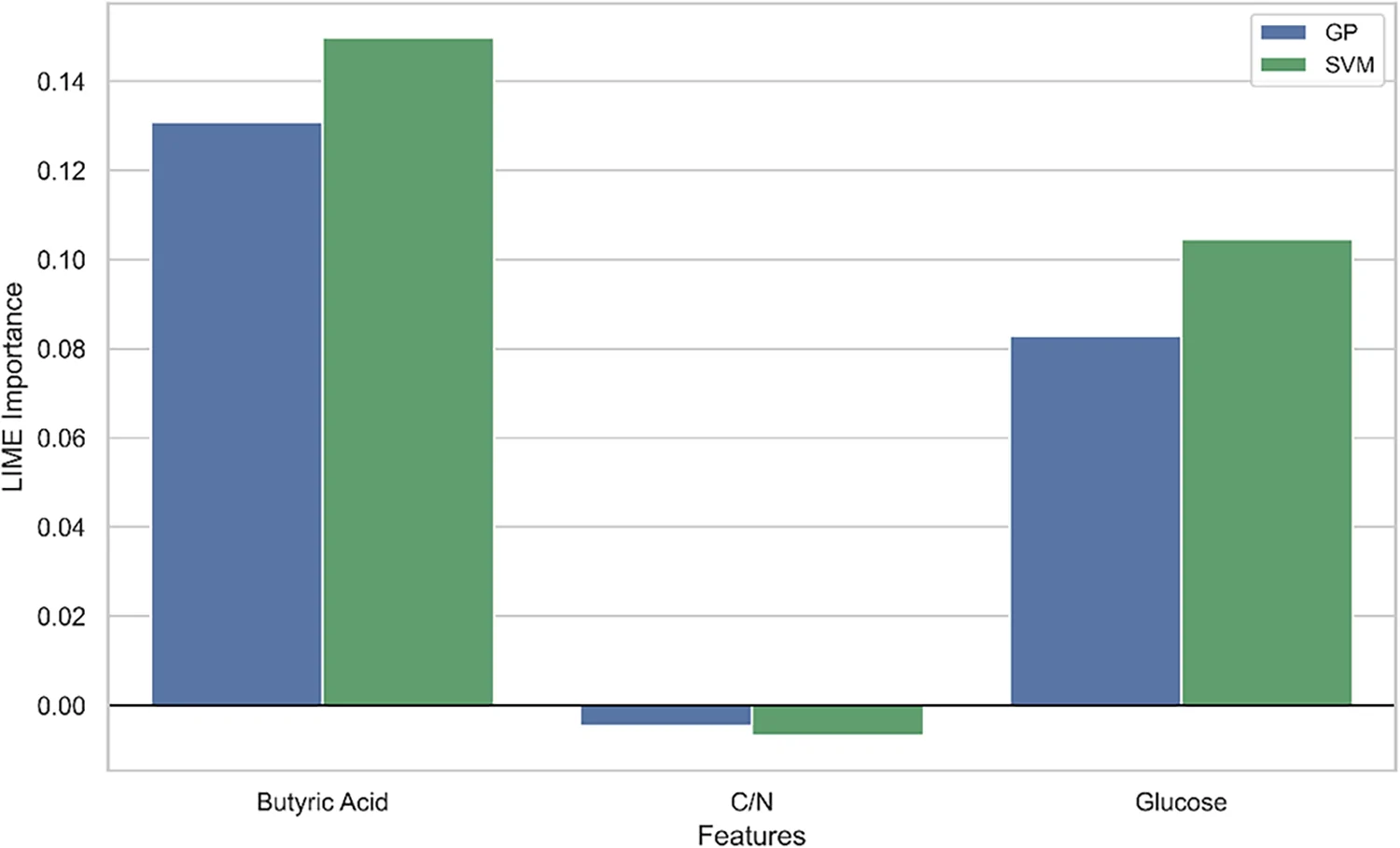
Average LIME importance for the features used for butanol prediction using both SVMs and GPs.

The LIME’s importance scores seen in Figure 6 corroborate the MI results shown in Figure 5, where the C/N ratio has little information and exhibits the lowest absolute value of LIME feature importance with respect to final butanol concentration, whereas glucose and butyric acid contain basically all the predictive power and display the highest LIME importance values. Moreover, the C/N ratio feature shows a negative contribution to the predicted butanol concentration, indicating that an increase in its value leads to a decrease in the model output. Therefore, models using the two key features achieve similar performance levels to those using all features, as seen in Table 2. These results are consistent with the ANOVA analysis performed for the polynomial model (Table S4), showing that only glucose and butyric acid have significance on the prediction.

Figure 7 presents a heatmap of testing RMSE when removing the different experiments from the training data set. Each row represents a candidate experiment to be removed, and the color indicates the testing RMSE obtained when that experiment is transferred from training to the testing set, in the same column. The experiment whose removal gives the lower testing RMSE (deep green) is permanently added to the testing set, then proceeds to the next column (step), which represents a new configuration of both data sets. The selected experiment is then removed from the heatmap. This process continues advancing to the next columns (5 times) and can be interpreted as, at each step, removing the experiment with the least unique information on the rest of the testing experiments, as performance capabilities are not affected.

**Figure 7:**
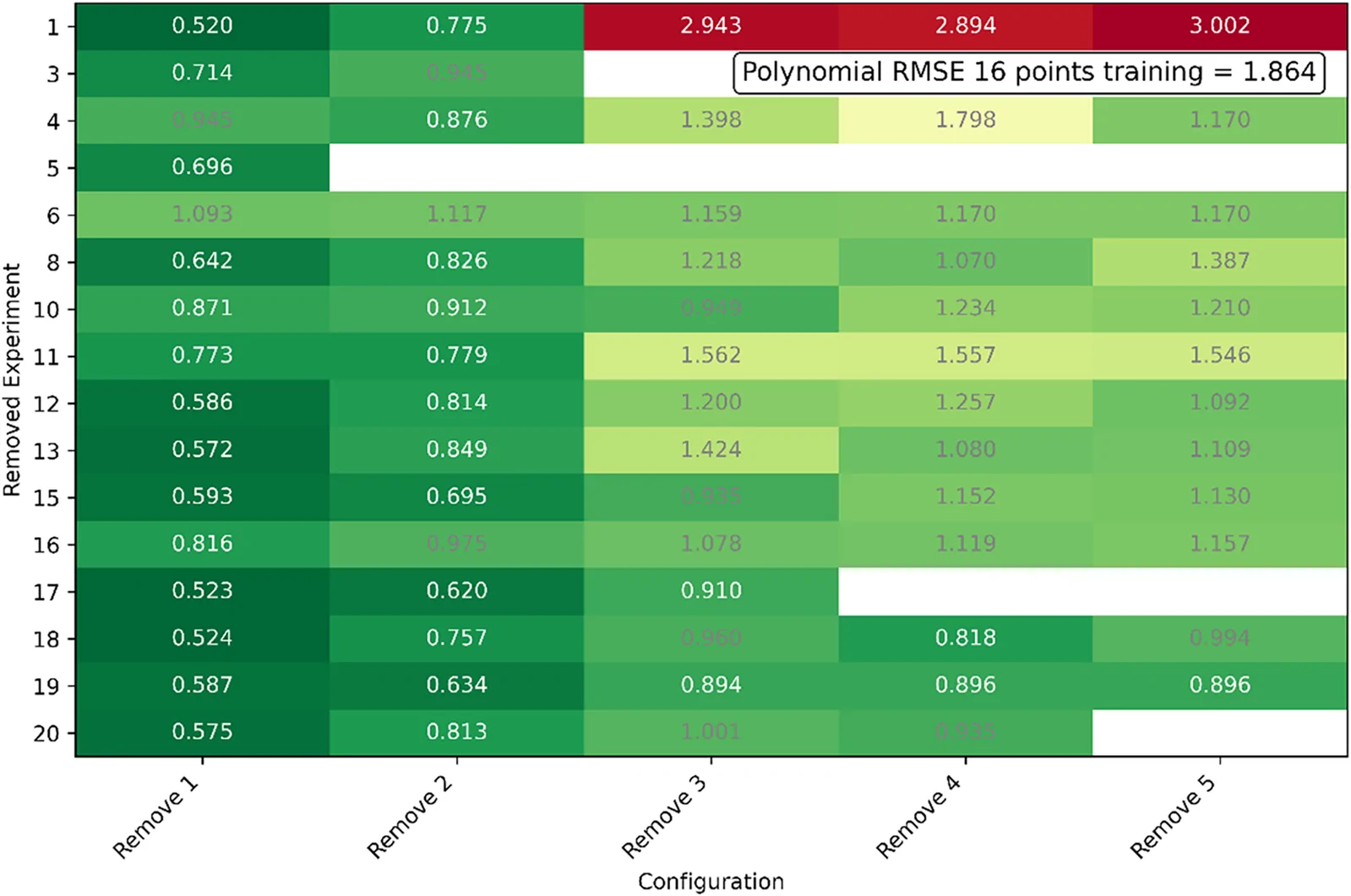
Heatmap of testing RMSE when sequentially removing experiments from training to testing data set using GPs.

Figure 7 shows that progressively removing experiments from the training data set toward the testing set deteriorates prediction capabilities, as expected. However, 93% of the evaluated schemes present better performance than the polynomial baseline, with 65% of the cases presenting better RMSE than the polynomial fitted with all the data. Interestingly, prediction capabilities collapse when removing Experiment 1 after removing Experiment 3 in the second round. This is because both experiments share the same conditions on Glucose and Butyric Acid (corresponding to one of the experimental design’s corners; see Table S2); as such, removing both results in worse performance in that region due to loss of fundamental information. On the other hand, RMSE for the GP in the first column of Figure 7 is 63% lower than the polynomial one, with prediction deteriorating to 56% lower in the second removed experiment, 32% in the third one, and around 30% for the fourth and fifth removal.

Complementary, Figure S1 presents the same information as Figure 7, but using full polynomial quadratic models instead of GPs. These polynomial models present worse performance than all ML models, with only 3 out of 70 presenting better or equal performance than when training with 16 experiments. Moreover, RMSE deteriorates on average by 83.33%, with cases having up to 5x higher RMSE in validation sets.

To provide deeper insights into this analysis, Figure S2 shows the evolution of Mahalanobis distances^52^ between the removed experiment and the training and testing feature distributions along the optimal removal sequence, defined as the sequence that maximizes testing RMSE. Each point corresponds to one removal round, and the arrows indicate the progression order. The first removed experiment exhibits a large Mahalanobis distance to the training distribution and a much smaller distance to the testing distribution, suggesting that it is less representative of the training set than of the testing set. As the sequence progresses, both distances decrease, and the points move toward the lower-distance region, indicating that the removed experiments become more typical of both distributions. Figures S3 and S4 present a conceptual 2D interpretation of this phenomenon: feature coordinates closer to the training and testing data sets’ distributions are presented in Figure S3, while Figure S4 shows a given coordinate close to the mean value of both distributions. In Figures S3 and S4, orange and blue dashed ellipses represent constant Mahalanobis distance contours, enclosing approximately 87% of the points in each design space distribution. Results in Figure S2 indicate that after removing the third experiment, the feature combination is nearly equally representative of both sets, with training and testing data sets having similar centers and covariance structures, as schematically shown in Figure S4. Consequently, exchanging one experiment for another produces little additional distributional shift. This behavior is consistent with the reduced deterioration in testing RMSE observed in Figure 7, since later removals appear less influential, evidenced by average RMSE being around 30% lower from the third experiment removed onward.

Figure 8 presents the MAPE on the identified optimal conditions (validation set) after removing the experiments. The lower the MAPE, the darker and bigger the bubble. In contrast to the results on validation RMSE, ML models exhibited lower MAPE on all instances. This is explained because the optimal conditions are centered on the design space (Table S2), and removing information on the corners (Experiments 1 and 3) does not affect the prediction significantly. When comparing the polynomial fitted on all data, 87% of the ML models exhibited better MAPE.

**Figure 8:**
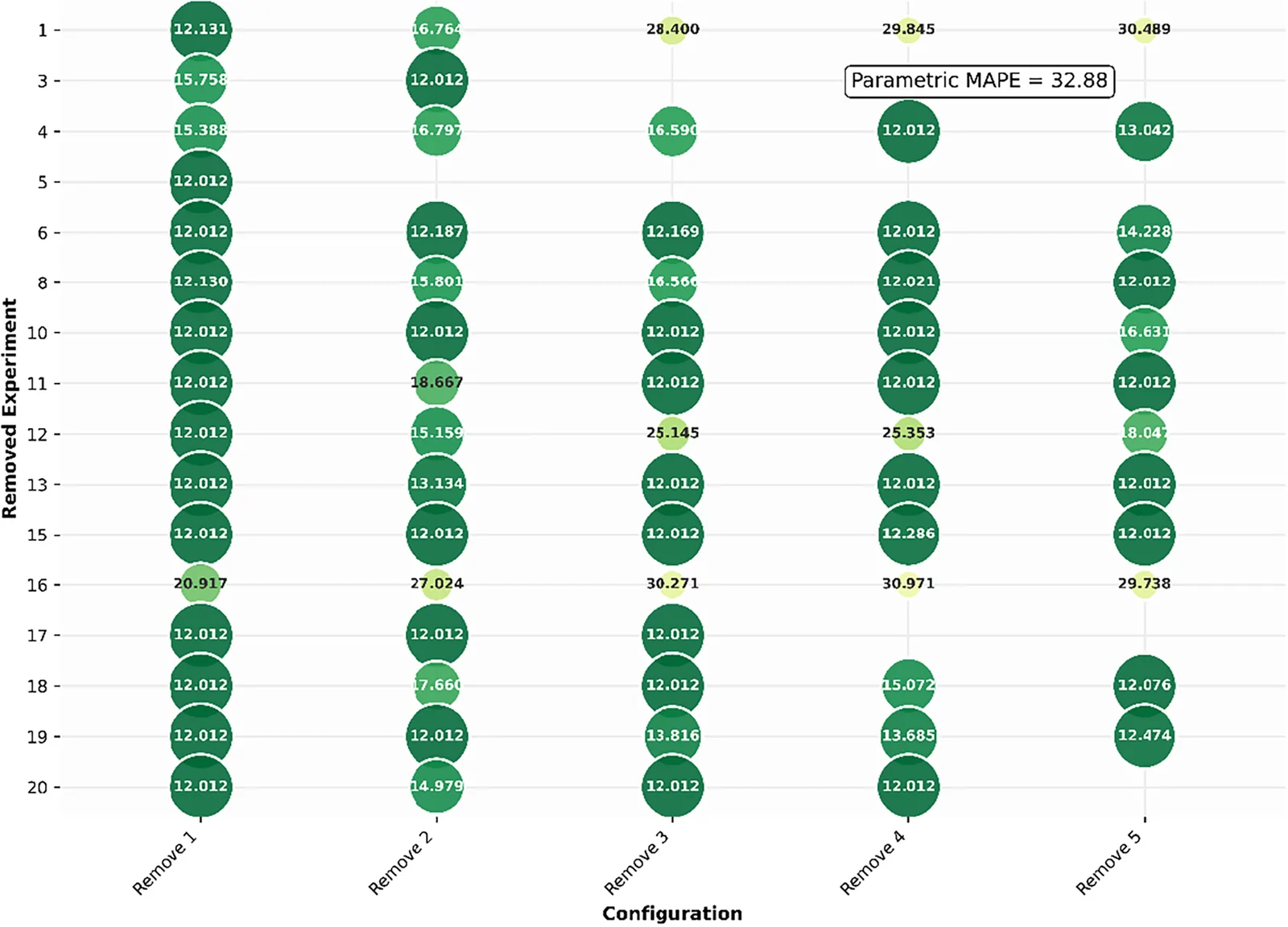
MAPE with regard to optimal conditions when removing successive experiments from the training set.

Like in the previous case, ML models exhibited superior performance to the polynomial models, while using less information (one less feature). Moreover, most of the ML models retained higher performance in testing and validation sets when compared against the polynomial approach, despite only using half of the experiments for calibration.

### 3.3 Case 3: Model-Based DoE (MBDoE)

Using the original kinetic model for continuous aerobic fermentation for *S. cerevisiae* with overflow metabolism to generate synthetic data,^39^ initial experimental system responses were provided using Factorial Designs and LHD to systematically explore the design space in a sequential MBDoE. Initial data points consisted of 4, 9, and 16 data sets, with feeding substrate concentration between 30 and 60 g/L and dilution rate between 0.35 h^–1^ and 0.5 h^–1^ (this region includes the multiple steady states). Afterward, this initial set of experiments was used to calibrate different ML and polynomial models. Using those models, optimal conditions for ethanol productivity were predicted, and the experiment was simulated using the original model to corroborate the RSM prediction. This predicted optimum was added to the calibration set, and models were recalibrated to again predict the optimum. This process was repeated until the ML/polynomial models’ prediction of the optimal conditions did not change. Table 3 presents the performance of the different models tested in the MBDoE approach.

**Table 3:** Performance of the Different Models in the MBDoE, Including Number of Experiments and MAPE for Both the True Optimal Conditions of Critical Dilution Rate and Obtained Ethanol Productivity

				MAPE	MAPE
type of model	type of DoE	initial points	extra experiments	dilution rate	ethanol productivity
Polynomial	Factorial	9	7	26.35%	25.10%
Polynomial	Factorial	16	10	21.63%	22.45%
Polynomial	LHD	9	10	14.63%	25.80%
GP	Factorial	4	7	12.68%	16.47%
GP	Factorial	16	3	7.48%	9.24%
GP	LHD	4	6	10.59%	9.68%
GP	LHD	9	2	1.59%	0.02%
SVM	LHD	9	2	5.13%	0.03%

Table 3 shows that ML models outperformed the polynomial approach, yielding lower MAPE values for both optimal ethanol productivity and the critical dilution rate. The observed MAPE reductions ranged from 26.6% to 99.9% and from 27.6% to 89.1%, respectively. Substrate feeding concentrations were not used for comparison as the original kinetic model does not incorporate substrate inhibition terms and all models converged to the experimentally tested upper limit of 60 g/L. The results depict how using factorial experimental designs as a basis for MbDoE yielded poorer performance when compared to LHD designs, even when initially they had almost twice as many experimental data points as the LHD (16 experimental points vs 9). This suggests that for complex nonlinear systems (as bioprocesses), LHD designs may be better suited to efficiently explore the design space. Likewise, GPs and SVMs achieved MAPE lower than 1% for the target variable when using only 9 initial data points, signaling better generalization capabilities even with sparse data.

Figure 9 shows the evolution of MAPE for both dilution rate (top) and ethanol productivity (bottom) as a function of additional experiments. When using only 4 initial LHD points for calibrating GP models, there are required the most additional experiment (7) for the model to converge to a single prediction. On the other hand, when using 9 initial LHD experiments, both GP and SVM models converge with three extra points. Interestingly, using a Factorial Design with 16 points needs 4 additional experiments to converge. However, its performance is the worst on the target variable.

**Figure 9:**
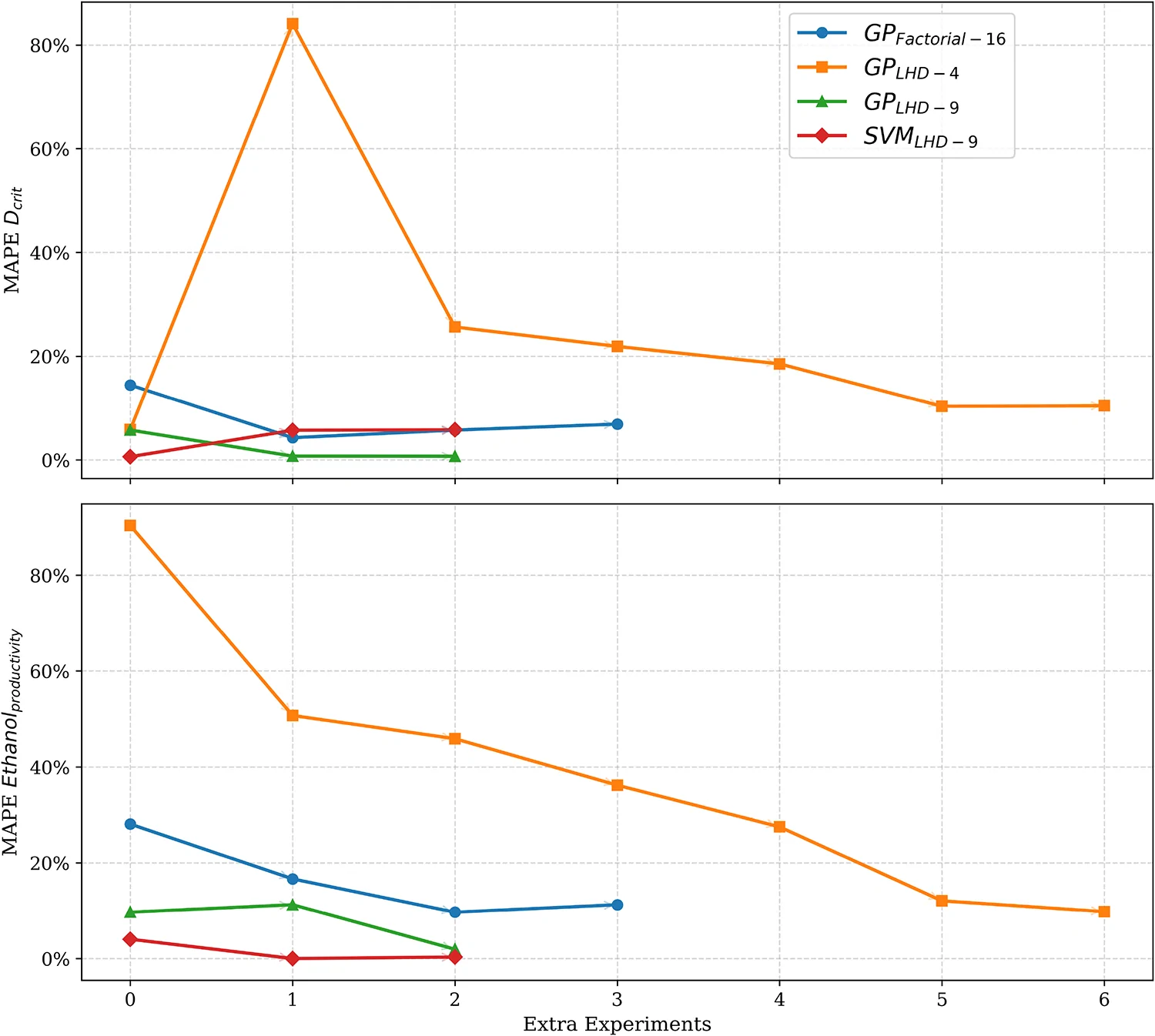
MAPE of the different ML models as a function of the additional experiments. Extra experiments start at 0 to signal the optimal point predicted by the initial DoE.

Figure 10 presents the phase plane of different trajectories followed by the ML models along with the additional experiments needed. As seen, ML models tend to converge in both response variables, lowering error and approaching the utopia point at (0,0). Interestingly, there seems to be a trade-off between the initial number of experiments, additional runs, and MAPE. Using factorial designs with 16 points as a starter yielded 3 additional runs, but MAPE was higher than using an LHD with only 4 initial points, despite it taking as many as 6 experiments to converge. Likewise, this LHD yielded the least total number of experimental runs, but not the best MAPE overall. On the other hand, using 9 initial LHD points granted the least number of additional required runs (2), and despite not being the lowest number of experiments, both GP and SVM models presented the best performance.

**Figure 10:**
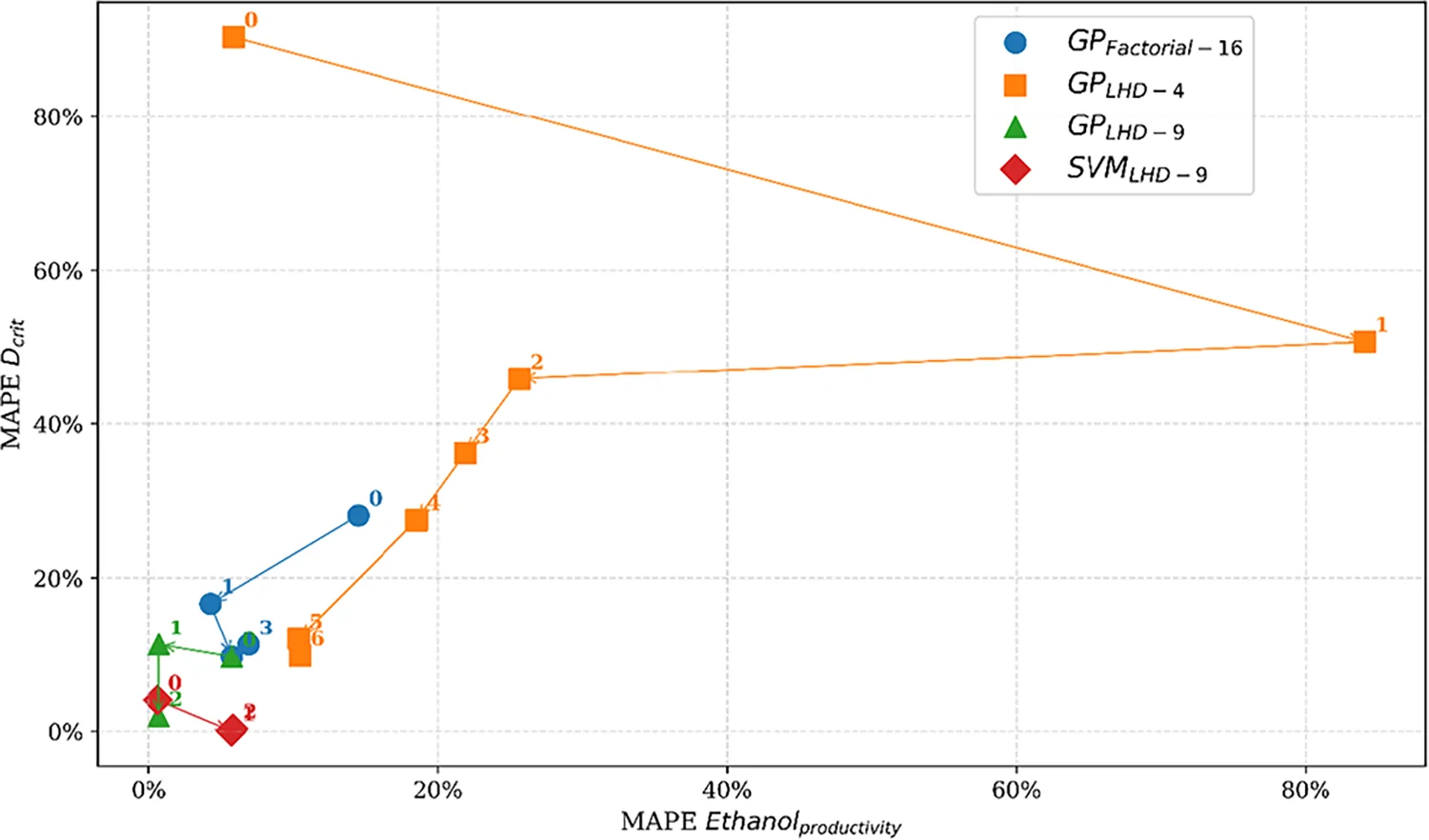
Phase plane trajectories of the different ML models with the additional experiments performed.

These results show that ML models used in an MBDoE setup presented higher performance when searching for optimal conditions in the design space than polynomial models. Despite factorial approaches having more initial information, LHD designs enhanced by ML models (SVMs and GPs) presented fewer experimental runs and higher accuracy.

## 4 Conclusions

This work utilized a computational proof of concept showing that ML models exhibit enhanced predictive capabilities compared to traditional polynomial models within the response surface method, both in calibration and validation sets for different problems during the early stage of experimental design, which was expected for nonlinear bioprocess behavior. However, the ML models retained superior performance even when being calibrated with less information (number of experiments or less features). Techniques such as MI and CMI were effective in selecting relevant features for the target variables. MI’s effect was corroborated using LIME to explain how the ML models make predictions, making them comparable to the interpretability by design of polynomials. Moreover, it was shown that if a feature was predicted to have little to no information on the target variables, LIME scores showed ML models not relying on them to make predictions. This agreed with the traditional significance of factors used in ANOVA. Nonetheless, MI and LIME results should not be interpreted as biological causalities or mechanistic explanations.

A MBDoE approach using synthetic data on ethanol fermentation showed potential applications of ML models on accelerating bioprocesses early stage exploration. ML models using LHD approaches predicted target variables with less than 1% error, while traditional polynomial models could not reach errors lower than 16%, despite being calibrated with as many as three times the experimental runs.

Individual experimental runs studied in this contribution take from 12 h for succinic acid production,^37^ 140 h for butanol fermentations,^38^ and 24 days of continuous experiments for ethanol production.^39^ As such, this proof of concept indicates that ML models might help reduce the number of experimental trials, thereby potentially lowering both the time required for experimentation and the associated costs (e.g., reagents, equipment, energy, personnel), as demonstrated in Butanol’s case study, where 9 experiments were found redundant.

Furthermore, since ML techniques are not constrained by parametric (polynomial) models, they can effectively capture the nonlinear behavior of variables, which enhances the predictive performance of the models. These results indicate that ML models might enhance RSM and reduce experimental burden, thus accelerating bioprocess development. Moreover, incorporating techniques such as MI and LIME for feature selection and explainability mitigates the “black-box” component of ML models, making them suitable for extracting mechanistic insight. As three different cases were studied, the methodology might be transferable to other biotechnological systems. Likewise, results show that ML models can potentially be beneficial in highly nonlinear cases, such as complex bioprocesses. Moreover, further work should be done to assess whether ML models calibrated using this approach can be transferred to other bioprocesses. However, these results have been tested computationally with both real experimental data and synthetic data; for full validation, the framework should be applied to novel DoEs and bioprocess development. Furthermore, this methodology can assist early stages of bioprocess development, although the implementation should be explored in a case-by-case basis.

Finally, this framework pushes bioprocess optimization and development toward a more efficient and sustainable approach, aligned with core principles of Green Chemistry and Engineering:

- **Waste Prevention and Energy and Utility Efficiency**: Given long cultivation timelines in the cases studied, a significant reduction in the number of experimental trials results in savings in energy consumption (associated with agitation, aeration, thermal regulation, and monitoring) as well as reductions in use and disposal of culture media, reagents, and solvents, thereby lowering the carbon footprint of process development.
- **Renewable Feedstocks**: By lowering data and temporal barriers for optimizing biobased systems utilizing renewable biomass, this framework enhances the viability and rapid deployment of sustainable alternatives designed to displace fossil-derived routes.

## Supplementary Material



## Notes

Experimental data, ANOVA for the polynomials used in Cases 1 and 2, RMSE in validation experiments for Case 2, and conceptual explanations of Mahalanobis distances (PDF)
