# Supplementary material for: Enhancing Response Surface Methodology for Bioprocesses Development Using Interpretable Machine Learning
Source: ACS Omega. 2026 Oct 29;11(44):69154–66. doi: 10.1021/acsomega.6c09217 (PMC13756668; doi:10.1021/acsomega.6c09217)
Supplement: Supplementary file 1 [file ao6c09217_si_001.pdf]

SUPPLEMENTARY MATERIAL FOR:

Enhancing Response Surface Methodology for Bioprocesses development using Interpretable Machine Learning

Juan Federico Herrera-Ruíz[a], Juan Camilo Castaño-Sánchez [b], Oscar Andrés Prado-Rubio*[c]

[a] *Grupo de Investigación en Aplicación de Nuevas Tecnologías (GIANT) Departamento de Ingeniería Química, Universidad Nacional de Colombia sede Manizales, Campus La Nubia, Manizales, 170003, Colombia*, email: jufherreraru@unal.edu.co

[b] *TBI, Université de Toulouse, CNRS, INRAE, INSA, 135 Avenue de Rangueil, Toulouse (CEDEX 04) 31077, France. email: jcastano-san@insa-toulouse.fr*

[c] *Grupo de Investigación en Aplicación de Nuevas Tecnologías (GIANT) Departamento de Ingeniería Química, Universidad Nacional de Colombia sede Manizales, Campus La Nubia, Manizales, 170003, Colombia* email.oaprador@unal.edu.co

*Corresponding author: Oscar Andrés Prado-Rubio; oaprador@unal.edu.co

SUPPLEMENTARY MATERIAL:

Table S1. Experimental conditions on the CCD for Succinic acid prediction. Taken from (Zhang et al., 2012).

Run	Glucose (g/L)	Yeast Extract (g/L)	$MgCO_3$ (g/L)	Succinic Acid (g/L)
1	15	5	15	7.884
2	30	5	15	10.403
3	15	10	15	11.692
4	30	10	15	15.127
5	15	5	30	3.26
6	30	5	30	6.223
7	15	10	30	12.601
8	30	10	30	18.863
9	9.89	7.5	22.5	6.782
10	35.11	7.5	22.5	17.804
11	22.5	3.3	22.5	0.104
12	22.5	11.7	22.5	14.998
13	22.5	7.5	9.89	11.226
14	22.5	7.5	35.11	16.831
15	22.5	7.5	22.5	14.447
16	22.5	7.5	22.5	15.435
17	22.5	7.5	22.5	15.07
18	22.5	7.5	22.5	17.425
19	22.5	7.5	22.5	18.541
20	22.5	7.5	22.5	17.518

Table S2. ANOVA for the Quadratic model based on the CCD for Succinci Acid production. Taken from (Zhang et al., 2012). A: Glucose Concentration. B: Yeast Extract concentration. C: $MgCO_3$ concentration. Significance level: 95%,

**Model term**	**Estimated Value**	**F value**	**P value**
Independent Term	16.41	-	-
A	2.47	22.11	0.0008
B	4.07	60.03	<0.0001
C	0.39	0.54	0.4796
AB	0.53	0.59	0.4602
AC	0.41	0.36	0.5645
BC	1.68	6	0.0342
A^2	-1.5	8.63	0.0149
B^2	-3.18	38.65	<0.0001
C^2	-0.89	3.02	0.1131

Experimental Data for Butanol Case

Table S3. Experimental conditions on the CCD for Butanol prediction. Taken from (Al-Shorgani et al., 2018)

Run	Glucose (g/L)	C/N ratio (g/g)	Butyric acid (g/L)	Butanol (g/L)
1	20	10	0	4.85
2	80	10	0	10.66
3	20	120	0	3.01
4	80	120	0	11.11
5	20	10	10	7.25
6	80	10	10	8.94
7	20	120	10	6.64
8	80	120	10	10.95
9	5	65	5	0.9
10	100.5	65	5	11.5
11	50	5	5	11.18
12	50	157.5	5	9
13	50	65	0	9.23
14	50	65	13.4	10.41
15	50	65	5	10.89
16	50	65	10	12.16
17	50	65	5	10.64
18	50	65	5	10.53
19	50	65	5	9.9
20	50	65	5	10.08
Identified Optimal	50	65	10	13.82

ANOVA for Quadratic model

Butanol

Table S4. ANOVA for the Quadratic model based on the CCD for Butanol prediction. Taken from (Al-Shorgani et al., 2018) A: Glucose Concentration. B: C/N ratio. C: Butyric Acid concentration. Significance level: 95%,

Model term	Estimated Value	F-value	p-value
**Independent Term**	-0.838	-	-
**A**	0.294	143.497	< 0.0001
**B**	0.00431	0.0413	0.5651
**C**	0.571	8.248	0.0320
**AB**	0.000276	3.096	0.0837
**AC**	-0.00659	12.294	0.0114
**BC**	0.00127	1.531	0.3007
**A^2**	-0.00186	50.799	< 0.0001
**B^2**	-0.000195	4.698	0.2143
**C^2**	-0.00186	2.936	0.2235

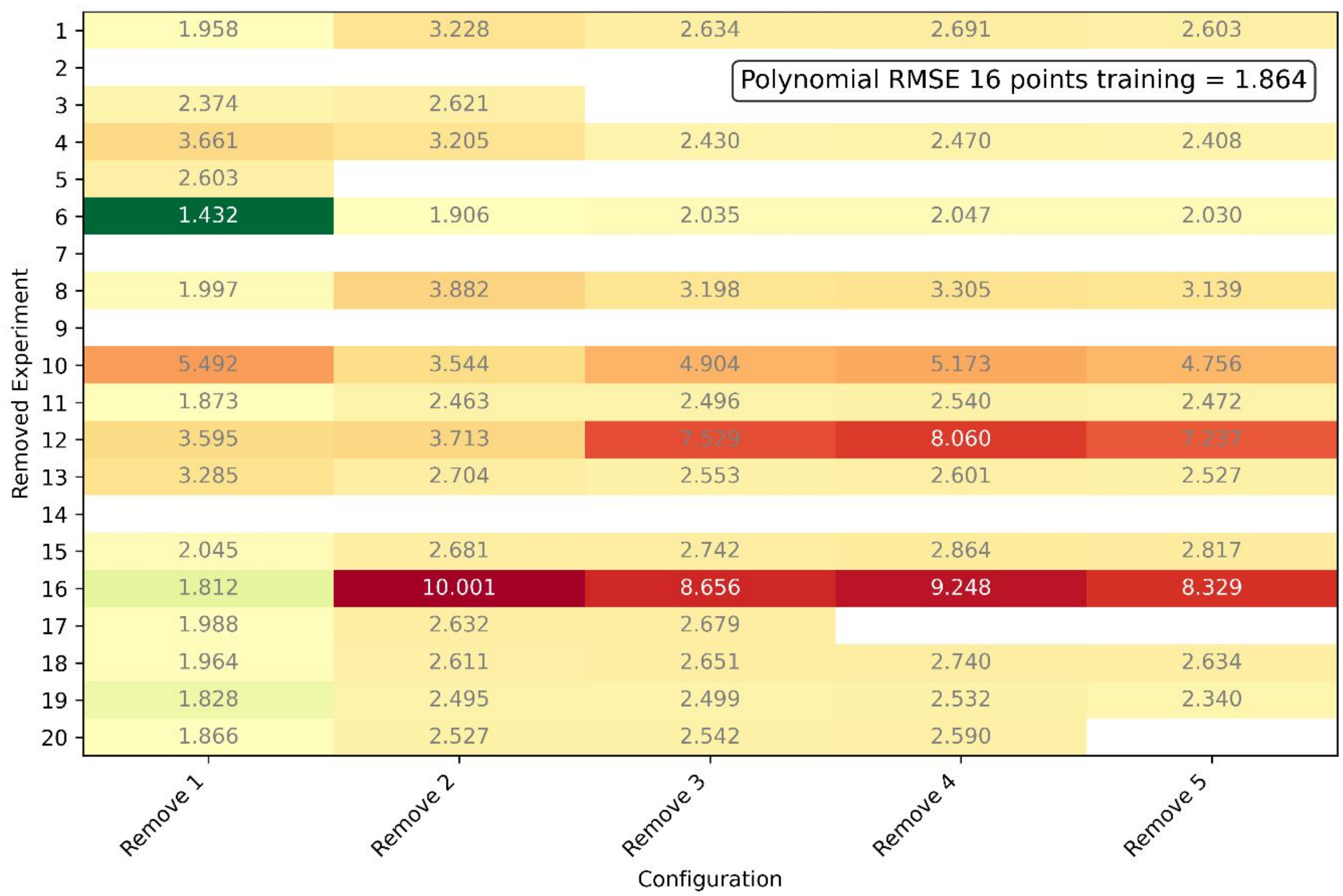

Figure S1. Heatmap of validation RMSE when sequentially removing experiments from training to validation data set using polynomial models.

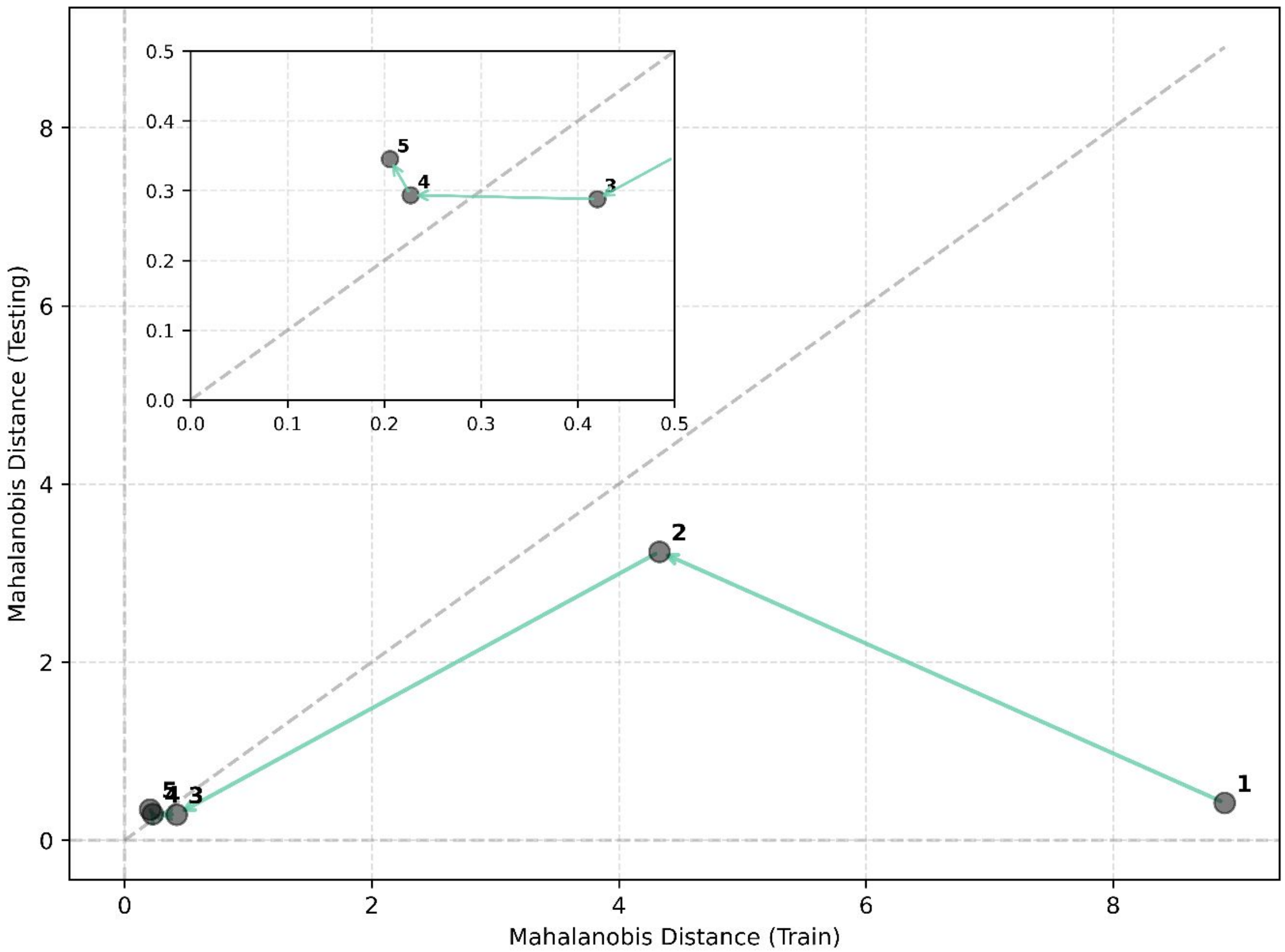

Figure S2. Mahalanobis distances for both training and validation sets when removing each experiments. The labels show the number of experiment removed, with the progression being. Experiments 5, 3,17, 20,19.

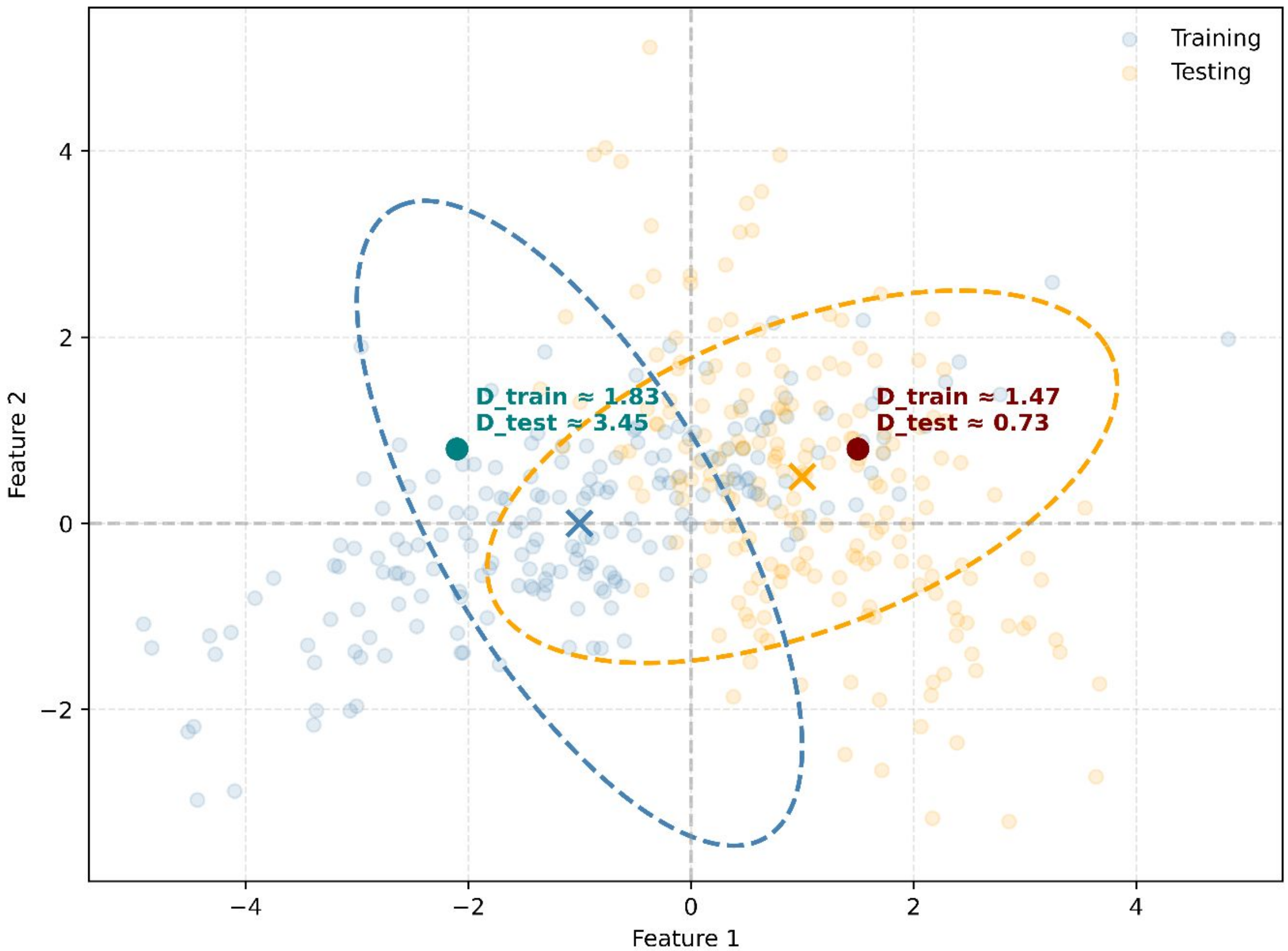

Figure S3. Conceptual explanation of Mahalanobis' distance for two given points to both training and validation sets. The crosses represent the mean for each distribution. Teal point is closer to the training distribution. Maroon point is closer to the testing distribution.

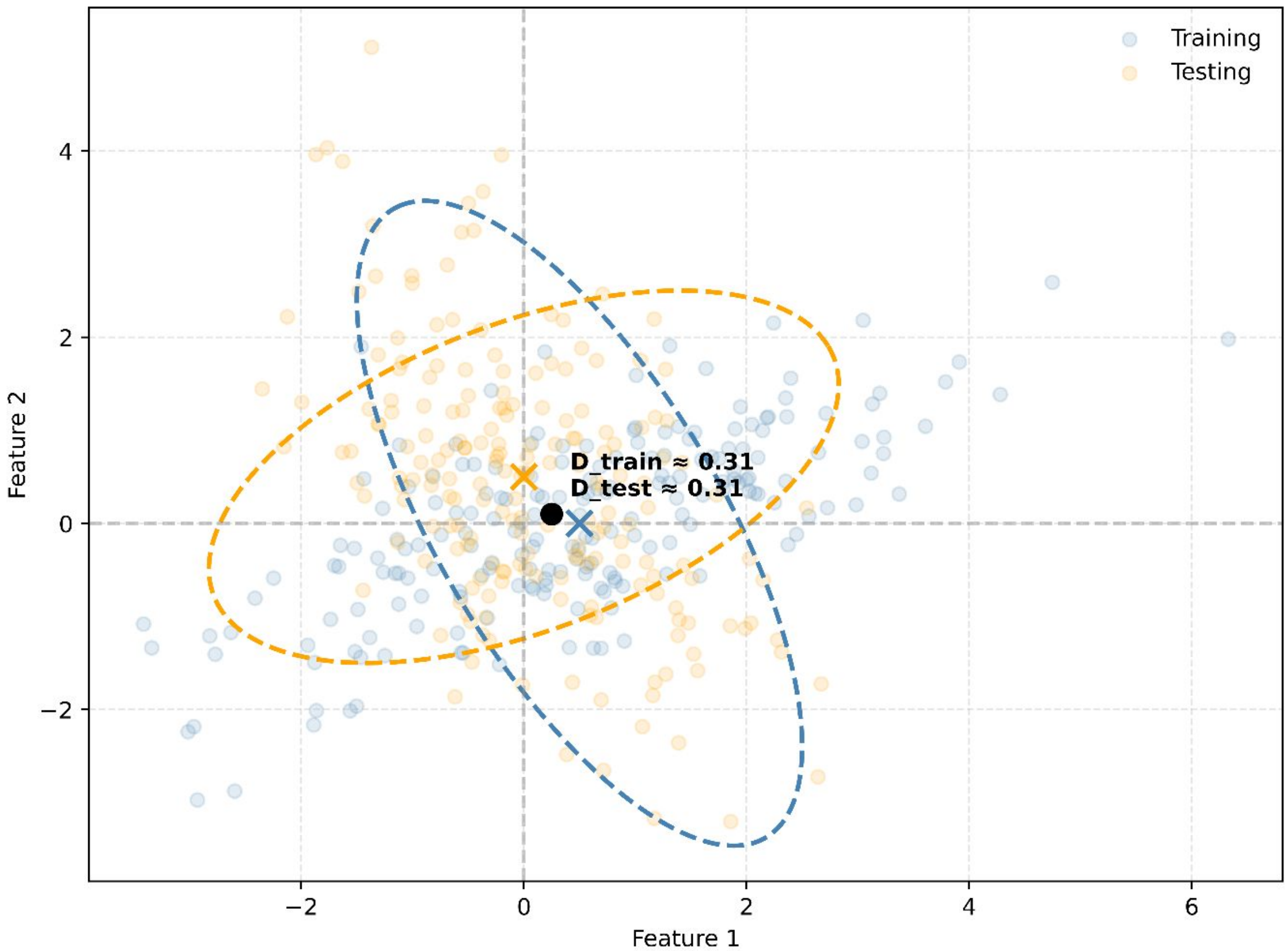

Figure S4. Conceptual explanation of Mahalanobis' distance for a given point to both training and validation sets. The crosses represent the mean for each distribution. In this case, the given point is close to the mean for both distributions.

ADDITIONAL INFORMATION ON CALCULATION DETAILS.

All Machine Learning models were trained using standard MATLAB functions in the Statistics and Machine Learning Toolbox. Hyperparameter optimization was performed using the standard Bayesian optimization methods found in MATLAB's *bayesopt* function.

Some of the ML model hyperparameters for each case are listed here:

1. **Case 1**
    a. SVMs using full inputs: Gaussian Kernel, and Kernel Scale 0.1168.
    b. SVMs using reduced dataset: Polynomial Kernel, and Kernel Scale 1.
    c. GPs using full input features: ARDMatern32 Kernel, and Linear Basis Function, estimated noise of the predictor: $1.55*10^{-4}$.
    d. GPs using reduced input features: ARDSquareExponential Kernel, and Linear Basis Function, estimated noise of the predictor: $2.78*10^{-4}$.
2. **Case 2**
    a. SVMs using full inputs: Gaussian Kernel, and Kernel Scale 86.1568.
    b. SVMs using reduced dataset: Gaussian Kernel, and Kernel Scale 96.0480.
    c. GPs using reduced input features: ARDSquareExponential Kernel, and PureQuadratic Basis Function, estimated noise of the predictor: $1.03*10^{-4}$.
    d. GPs using full input: ARDRationalQuadratic Kernel, and Linear Basis Function, estimated noise of the predictor: $1.12*10^{-4}$.
3. **Case 3**
    a. GP starting with 4 experiments and Factorial Design: SquaredExponential Kernel, and Constant Basis Function, estimated noise of the predictor: 0.9348.
    b. GP starting with 16 experiments and Factorial Design: SquaredExponential Kernel, and Constant Basis Function, estimated noise of the predictor: 0.5372.
    c. GP starting with 9 experiments and LHD: SquaredExponential Kernel, and Constant Basis Function, estimated noise of the predictor: 0.2365.
    d. GP starting with 4 experiments and LHD: SquaredExponential Kernel, and Constant Basis Function, estimated noise of the predictor: 0.2219.
    e. SVM starting with 9 experiments and LHD: Linear Kernel, and Kernel Scale 0.015.
